# BMP9 promotes cutaneous wound healing by activating Smad1/5 signaling pathways and cytoskeleton remodeling

**DOI:** 10.1002/ctm2.271

**Published:** 2021-01-15

**Authors:** Peiwei Chai, Jie Yu, Xi Wang, Shengfang Ge, Renbing Jia

**Affiliations:** ^1^ Department of Ophthalmology Shanghai Key Laboratory of Orbital Diseases and Ocular Oncology Ninth People's Hospital Shanghai Jiao Tong University School of Medicine Shanghai P.R. China

**Keywords:** animal model, BMP9, wound healing

**ABBREVIATIONS**
BMP9: bone morphogenetic protein‐9
CDS: coding sequence
COL: collagen
ECM: extracellular matrix
GAPDH: glyceraldehyde 3‐phosphate dehydrogenase
GDF2: growth and differentiation factor 2
HA: hyaluronic acid
NC: negative control
p‐Smad: phosphorylated Smad
siRNA: small interfering RNA
TGF‐β: transforming growth factor‐beta
α‐SMA: alpha smooth muscle actin

Dear Editor,

Herein, we revealed that bone morphogenetic protein‐9 (BMP9) serves as a novel accelerator in cutaneous wound healing. When cutaneous injuries occur, highly integrated cellular and molecular events are programmed to guarantee the proper and timely healing of wounds.^1^, ^2^ Pathological wound healing is characterized by delayed and impaired wound closure and eventually facilitates infection and chronic inflammation, specially in eyelid and limbs.^3^, ^4^ Although many regulatory elements of wound healing have been identified, the role of BMP9 signaling in skin injury repair remains unclear.

Therefore, we aimed to define the role of BMP9 in wound healing. Bmp9‐deficient and wild‐type mice were genotyped by PCR (Figure 1A) as described.^5^, ^6^ Through H&E staining analysis of dorsal skin samples, we discovered that Bmp9 knockout mice presented a thinner corium layer than wild‐type (WT) controls (Figures 1B and 1C). To explore whether BMP9 regulates cutaneous wound healing, we established a skin injury model with a 4‐mm‐diameter punch on the dorsal skin of Bmp9 knockout and WT mice. We found that the wound closure rate significantly slowed in Bmp9 knockout mice compared with WT mice (Figures 1D and 1E). In addition, we observed a marked inhibition of re‐epithelization in the wounded areas of Bmp9‐deficient mice in contrast to that in the WT controls (Figure 1F). We also observed significantly decreased circulating BMP9 levels in patients with diabetic foot, while there were no significant differences in serum Bmp9 levels between unaffected patients and patients with diabetes without chronic wounds in either the type 1 diabetes or type 2 diabetes group (Table S1, Figure 1H).

**FIGURE 1:**
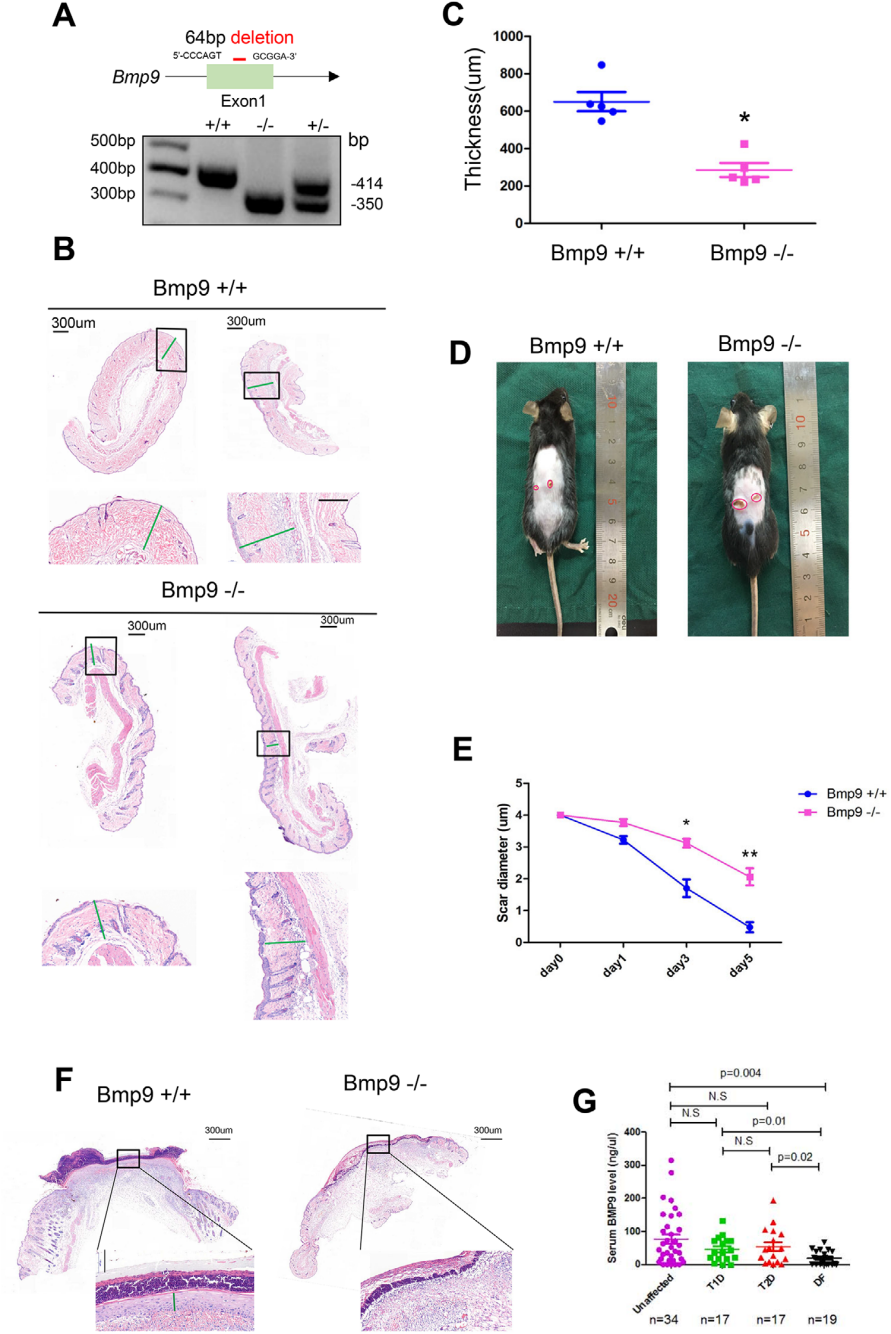
Bmp9 deficiency leads to delayed wound healing in vivo. A, Genotype of Bmp9−/−, Bmp9−/+, and Bmp9+/+ mice. B, The cutaneous thickness was measured in both Bmp9−/− and Bmp9+/+ mice by H&E staining of the dorsal skin; scale bar: 300 μm in the upper panel and 100 μm in the lower panel. C, The cutaneous thickness was measured in Bmp9−/− and Bmp9+/+ mice. The results indicated that Bmp9 knockout mice presented a thinner corium layer than Bmp9+/+ mice. n = 5 for each group, *P < .05. D, A 4 mm wound was created in both Bmp9−/− and Bmp9+/+ mice. E, The average wound diameter was measured in Bmp9−/− and Bmp9+/+ mice. The wound closure rate is significantly decreased in Bmp9 knockout mice. Average wound diameter = (long + short diameters)/2. n = 5 for each group. *P < .05, **P < .01. F, H&E staining of the wound area in Bmp9−/− and Bmp9+/+ mice. Significant inhibition of re‐epithelization in the wounded area in Bmp9‐deficient mice was observed. The green line represents the re‐epithelization. Scale bar: 300um. G, The serum BMP9 level in patients with unaffected (normal control, n = 34), type 1 diabetes (T1D, n = 17), type 2 diabetes (T2D, n = 17), and diabetic foot (DF, n = 19). We observed a significantly decreased circulating BMP9 levels in patients with diabetic foot (DF) while there were no significant differences of serum Bmp9 level between unaffected patients and diabetes without chronic wound patients, either in T1D or T2D group

The scratch assay showed that BMP9 treatment dramatically accelerated the healing rate of human dermal fibroblasts (HDFs) after sterile micropipette injury (Figures 2A and 2B). In parallel, BMP9 stimulation promoted the in vitro wound healing of a human keratinocyte cell line (HaCaT) (Figures 2C and 2D). In particular, the migratory capacity of HaCaT cells treated with BMP9 was enhanced, as determined by the Transwell assay (Figures 2E and 2F). We found that BMP9 treatment did not boost the proliferation of HDFs (Figure S1A), while it strongly induced the growth of HaCaT cells, as detected by either the CCK‐8 assay (Figure S1B) or colony formation assay (Figures 2G and 2H). Taken together, BMP9 promotes in vitro wound healing through its effects on fibroblast migration and keratinocyte proliferation and migration.

**FIGURE 2:**
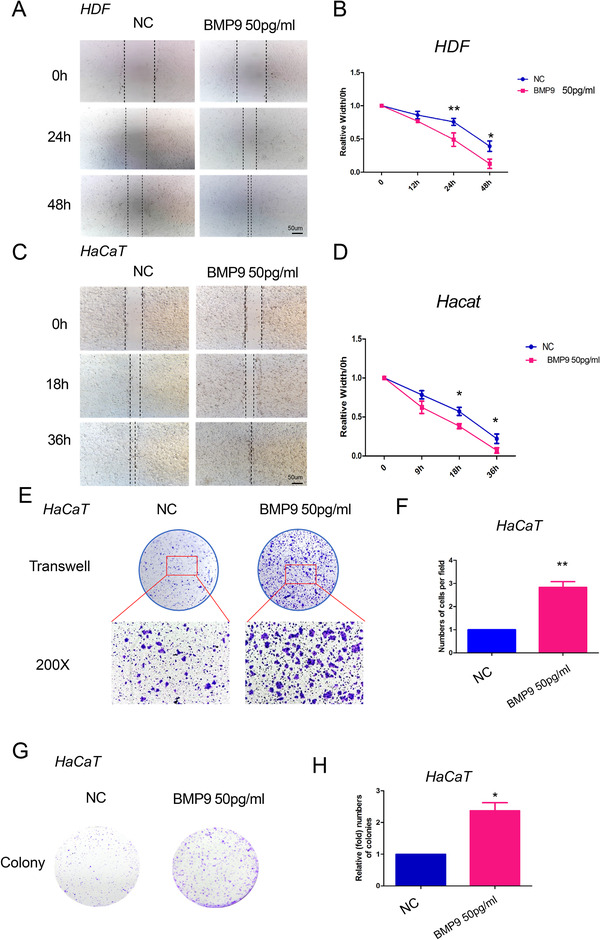
BMP9 promotes the migration of fibroblasts and keratinocytes. A and B, A wound healing assay was performed in human dermal fibroblasts (HDFs) with and without exogenous BMP9. BMP9 stimulation significantly promoted the migration of HDFs. *P < .05, **P < .01. C and D, A wound healing assay was performed in HaCaT cells with and without exogenous BMP9. BMP9 stimulation increased the cellular migration ability of the keratinocyte cell line HaCaT. *P < .05, **P < .01. E and F, A Transwell assay was performed in HaCaT cells with and without exogenous BMP9. BMP9 stimulation increased the migration ability of HaCaT cells. *P < .05, **P < .01. G and H, A colony formation assay was performed in HaCaT cells with and without exogenous BMP9. BMP9 stimulation induced a significant promotion of cell growth in HaCaT cells. *P < .05, **P < .01

Next, we decided to examine the BMP9‐mediated molecular mechanisms underlying wound healing. We performed RNA‐seq analysis and label‐free protein quantification comparing BMP9‐treated and untreated HDFs, and we compiled the results to identify key regulatory pathways mediated by BMP9 (Figure 3A). Through gene set enrichment analysis, we found that fibroblast growth and the transforming growth factor‐beta (TGF‐β) signaling pathway were significantly upregulated in BMP9‐treated HDFs relative to control cells (Figure 3B). As key activating factors of the Smad signaling pathway, DNA‐binding protein inhibitor ID‐1 (ID1) and DNA‐binding protein inhibitor 2 (ID2) were significantly upregulated according to circle analysis (Figure 3C). We then investigated the molecular alterations in the TGF‐β signaling pathway in either HDFs or HaCaT cells in response to BMP9 treatment. As a result, elevated levels of Smad1/5 phosphorylation were observed after stimulation with BMP9 (50 pg/mL), while Smad 2/3 phosphorylation remained unchanged (Figure 3D). Moreover, BMP9 stimulation increased Smad1/5 phosphorylation levels in a dose‐ and time‐dependent manner (Figure 3E). In addition, we also observed decreased levels of ID1 and p‐Smad1/5 in the wound area of Bmp9‐/‐ mice (Figure 3F).

**FIGURE 3:**
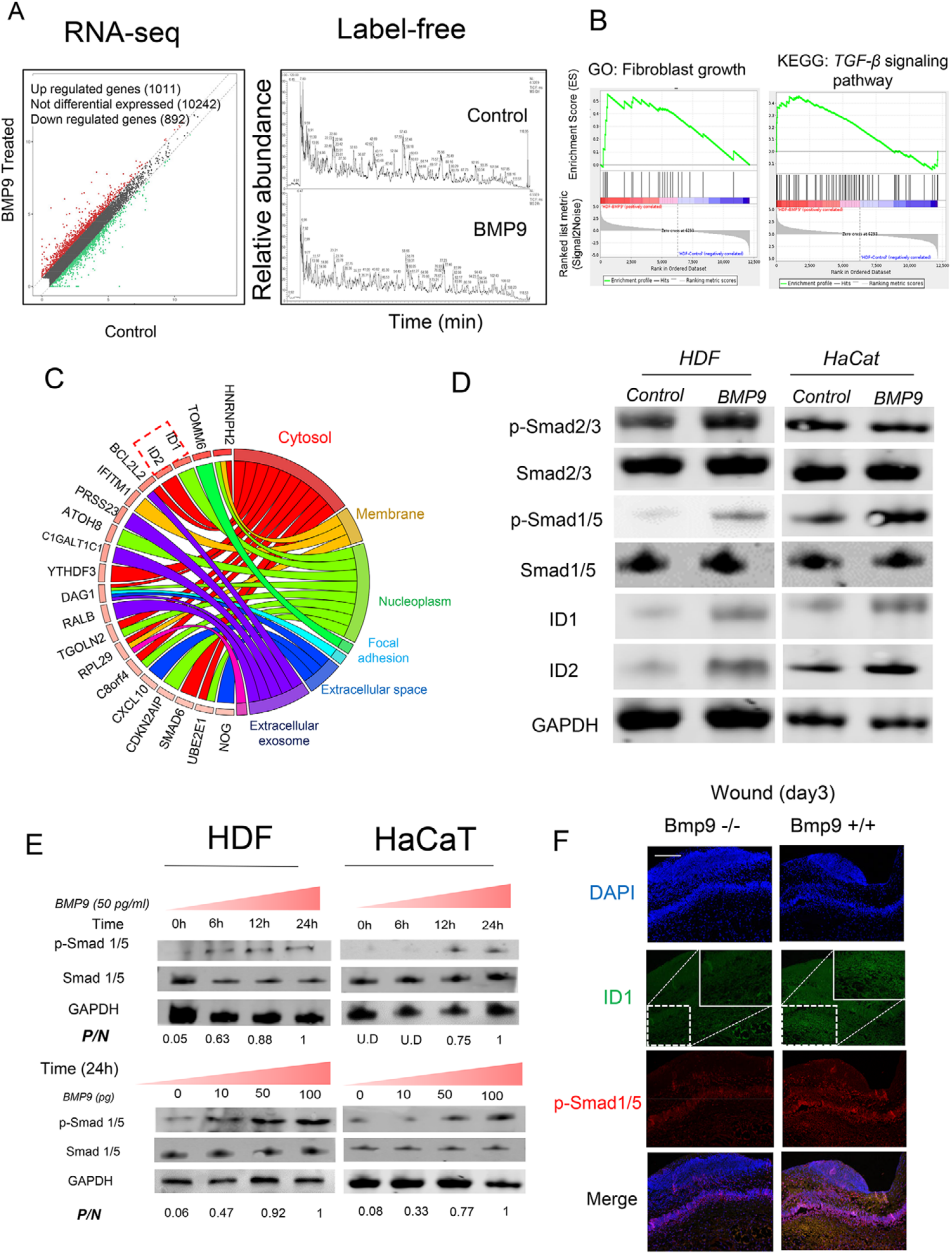
BMP9 activates the SMAD signaling pathway in fibroblasts and keratinocytes. A, HDFs were chosen as model cells and treated with and without recombinant human BMP9. RNA‐seq analysis and label‐free protein quantification comparing BMP9‐treated and untreated HDFs were performed. B, Gene set enrichment analysis (GSEA) showed that gene expression profiles associated with fibroblast growth and the TGF‐β signaling pathway were enriched in BMP9‐treated HDFs relative to control cells. C, A gene ontology (GO) circle analysis revealed key activating factors of the SMAD signaling pathway; ID1 and ID2 were activated. D, Western blotting was performed to measure the levels of Smad1/5 phosphorylation and ID1/2 after exogenous BMP9 treatment. Smad1/5 phosphorylation and ID1 and ID2 expressions were significantly increased following stimulation with 50 pg/mL BMP9, while Smad 2/3 phosphorylation remained unchanged. E, Western blotting was performed to measure Smad1/5 phosphorylation after exogenous BMP9 treatment in a dose‐dependent (0 pg/mL, 10 pg/mL, 50 pg/mL, and 100 pg/mL) and time‐dependent (0 hour, 6 hours, 12 hours, and 24 hours) manner. A weak Smad1/5 phosphorylation signal was observed without BMP9 administration. The level of Smad1/5 phosphorylation increased with the concentration of BMP9. The Smad1/5 phosphorylation level was highest in the presence of 100 pg/mL BMP9 and was comparable to that when the concentration of BMP9 was 50 pg/mL in both HDFs and HaCaT cells. In addition, the level of Smad1/5 phosphorylation also increased with treatment time. The Smad1/5 phosphorylation level was highest after 24 hours of stimulation with BMP9 at 50 pg/mL. F, Immunofluorescence of ID1 (green) and p‐Smad1/5 (red) was performed in the wound area in Bmp9−/− and Bmp+/+ mice. Decreased levels of ID1 and p‐Smad1/5 in the wound area of Bmp9−/− mice were observed. Scale bar: 300um. Abbreviations: P/N, the expression ratio of p‐Smad1/5 to native‐Smad1/5; U.D, undetected.

With further interpretation of the RNA‐seq and label‐free protein quantification data, we observed that BMP9 stimulation resulted in changes regarding cytoskeletal dynamics (Figures S2A and S2B). Many proteins that assist cell motility through the regulation of the cytoskeleton were upregulated after BMP9 stimulation of HDFs (Figure S2C). Furthermore, fibrosis‐related proteins, such as MYOSIN6, α‐SMA, and STMN2, were elevated after BMP9 stimulation, while collagen 1 was not disturbed (Figures S2D and S2E). Since STMN2 is strongly connected to dynamic cytoskeletal remodeling, we further checked and confirmed that the microtubule protein β‐tubulin was increased in the soluble supernatant of cell lysates after BMP9 stimulation (Figures S2E and S2F). In contrast, we found that F‐actin stress fibers displayed a sharp reduction in fibroblasts treated with BMP9 (Figure S2G). Collectively, these results demonstrate that BMP9 affects cytoskeletal remodeling to exert its influence on cell migratory capacity.

Given the essential role of BMP9 in a decent wound healing process, we next tested whether a replenishment of Bmp9 with Bmp9 ointment painted in the wound area could accelerate wound repair. Here, supplementation of Bmp9 in Bmp9‐deficient mice distinctly accelerated wound healing (Figures S3A‐S3C). For WT mice, we found that supplementation with Bmp9 accelerated the wound healing rate in the early phase (days 2‐4), while Bmp9‐treated or untreated WT mice showed almost the same healing rate when the wounds shrank to within 2 mm in diameter (Figures S3D‐S3F), suggesting that Bmp9 supplementation tends to interfere with early wound healing procedures.

Notably, Bmp9‐deficient mice exhibited cutaneous morphological changes, including a thinner corium layer. The major resident cell type of the dermis is dermal fibroblasts, which produce extracellular matrix and maintain the thickness of the corium layer.^7^ Herein, we observed significant activation in BMP9‐treated fibroblasts, with increased levels of extracellular matrix secretion, which provided an alternative explanation of the morphological changes in Bmp9‐deficient mice. In addition, we also observed a significant cytoskeletal change after BMP9 treatment. Importantly, a BMP9 auxiliary receptor, endoglin, has been proven to regulate actin cytoskeleton organization via zyxin and zyxin‐related protein 1,^8^, ^9^ which provides possible explanations for BMP9‐guided cytoskeleton remodeling.

Since circulating BMP9 can target endothelial cells, it is possible that BMP9 exerts its functions not only in fibroblasts and keratinocytes. Notably, our previous study revealed that the smooth muscle layer surrounding the vessel endothelial layer was fragile and discontinuous in Bmp9‐/‐ mice,^5^ which may lead to impaired angiogenesis during cutaneous wound healing. However, the comprehensive function of BMP9 in diversified cellular components during cutaneous wound healing requires further investigation.

In summary, we presented BMP9 as an accelerator of skin injury repair and provided a better understanding of the mechanism of wound healing. BMP9 directly stimulated the activation of dermal fibroblasts and keratinocytes through Smad signaling pathway activation and cytoskeleton remodeling to enhance skin repair (Figure S4).

## CONFLICT OF INTEREST

The authors declare that there is no conflict of interest that could be perceived as prejudicing the impartiality of the research reported.

## AUTHOR CONTRIBUTIONS

In this report, Renbing Jia, Peiwei Chai, and Jie Yu designed and performed the experiments and drafted the manuscript; Peiwei Chai, Shengfang Ge, and Renbing Jia were responsible for sample collection and data analysis; and Renbing Jia and Shengfang Ge discussed and revised the manuscript. Renbing Jia was the originator of the concept of this report and wrote and approved the manuscript. All authors approved this manuscript.

## Supporting information

Supporting Information
