# Supplementary material for: BMP9 promotes cutaneous wound healing by activating Smad1/5 signaling pathways and cytoskeleton remodeling
Source: Clin Transl Med. 2021 Jan 15;11(1):e271. doi: 10.1002/ctm2.271 (PMC7809598; doi:10.1002/ctm2.271)
Supplement: Supplementary file 1 — Supporting Information [file CTM2-11-e271-s001.pdf]

Supplementary files

BMP9 promotes cutaneous wound healing by activating Smad1/5 signaling pathways and cytoskeleton remodeling

Peiwei Chai[1*], Jie Yu[1*], Xi Wang[1*], Shengfang Ge[1], Renbing Jia[1],

[1]Department of Ophthalmology, Shanghai Key Laboratory of Orbital Diseases and Ocular Oncology, Ninth People's Hospital, Shanghai JiaoTong University School of Medicine, Shanghai, P. R. China

Shanghai 200025, China.

*These authors contributed equally to this report.

Corresponding author:

Renbing Jia, M.D., Ph.D., Ninth People's Hospital, Shanghai Jiao Tong University School of Medicine, Shanghai, 200025, P.R. China, e-mail: renbingjia@sjtu.edu.cn

KEYWORDS: BMP9, wound healing, animal model

RUNNING TITLE: The role of BMP9 in wound healing

MATERIALS AND METHODS

Animals

BMP9 wt and *BMP9 -/-* on the C57BL/6 background were maintained and bred under specific-pathogen-free conditions and genotyped. Ten- to 12-week-old male *BMP9 wt* mice and *BMP9 -/-* control littermates (offspring from heterozygote breeding) were used for the experiments as previously described(1). Genotyping and DNA sequencing results showed that 64 base pairs (bp) were deleted in first exon of BMP9 in BMP9-KO mice. We commissioned CYAGEN Bioscience Co., Ltd. to create the BMP9-KO mice by CRISPR/Cas-mediated genome engineering. The mouse BMP9 gene (GenBank accession number: NM_019506.4) is located on mouse chromosome 14. Mouse BMP9 contains 2 exons. Paired CRISPR gRNA1 (TGTACAAGTCGATCATGTACTGG) and gRNA2 (GCTGAAGCTCCGCACGATGTTGG) were designed and used to target sites in BMP9 exon 1. Cas9 mRNA and guide RNA (gRNA) generated by in vitro transcription were coinfected into fertilized eggs for KO mouse production. The positive pups were bred to the next generation, which was genotyped by PCR and DNA sequencing analysis. All mice were housed in a temperature-controlled room (at 22°C) with a 12-h light and dark cycle with free access to food and water. No remarkable change of gross appearance and life expectancy have been observed. Notably, we found the smooth muscle layer surrounding the vessel endothelial layer was fragile and discontinuous in Bmp9-/- mice(2). In addition, BMP9-KO mice exhibited a distinct physical appearance with a fatter body after approximately 16 weeks of common low diet feeding(1). The use of animals and animal-related experimental procedures were approved by the Institutional Laboratory Animal Care and Use Committee of Shanghai Ninth People’s Hospital.

RNA extraction, library construction and Illumina sequencing (RNA-seq)

Total RNA was extracted from the untreated and BMP9 treated HDF cells using TRIzol reagent (Invitrogen, Carlsbad, CA, USA). We confirmed the RNA integrity using the 2100 Bioanalyzer (Agilent Technologies, USA). We measured the RNA concentration in a Qubit 2.0 fluorometer by the Qubit RNA Assay Kit (Life Technologies, Carlsbad, CA, USA). We prepared the Libraries from 100 ng of total RNA using an Illumina TruSeq RNA Sample Prep Kit (San Diego, CA, USA) following the manufacturer’s protocol. Libraries were sequenced using the Illumina Hiseq 2500 platform (San Diego, CA, USA). The mRNA abundance levels of the unigenes identified using TopHat v2.0.9 and Cufflinks were normalized by the Fragments Per Kilobase of exon model per Million mapped reads (FPKM), and the log2 fold changes between two samples were tested statistically to determine whether an individual gene’s expression was altered significantly. We used the criteria of false discovery rate (FDR) < 0.01 and fold changes <0.5 or >2.0 (< −1 or >1 in log2 ratio value, P value<0.05) to identify the differentially expressed genes.

Tryptic digestion and peptide desalting for LC-MS/MS

First, 5 mM TCEP was added to each cell sample, which was mixed and incubated for 10 min. After the samples cooled to RT, 10 mM IAA was added followed by incubation in the dark for 15 min. Trypsin was resuspended with resuspension buffer to a concentration of 0.5 μg/μL and incubated at RT for 5 min. A 1-μL volume of trypsin solution was then added to each sample, which was mixed well, centrifuged,

and incubated with a Thermo mixer for 8 h. Then, the reaction was quenched with 1% TFA. C18 columns were equilibrated with 200 μL of acetonitrile (ACN). The ACN was then washed out with 200 μL of 0.1% FA 2 times, and the wash-out was discarded. The peptide solution was loaded into the tip of the C18 column and allowed to flow-through the column slowly; the flow-through was collected. The peptide loading step was repeated. Then, the column was washed with 200 μL of 0.1% FA, and the wash-out was discarded. The peptides were eluted with 50 μL of 70% ACN, and the eluate (B) was collected in a new tube. The desalting procedure was repeated (up to the 6th step) using the flow-through (A). The 2 eluates (B) were combined and vacuum dried. The peptides were resuspended with 10 μL of 0.1% FA for LC-MS/MS analysis or stored as a peptide powder at -80°C.

LC-MS/MS

Half of each peptide sample was separated and analyzed with a Nano-HPLC coupled to a Q-Exactive mass spectrometer (Thermo Finnigan). Separation was performed using a reversed-phase column (100 µm, ID × 15 cm, Reprosil-Pur 120 C18-AQ, 1.9 µm, Dr. Math). The mobile phases were H_2O with 0.1% FA and 2% ACN (phase A) and 80% ACN and 0.1% FA (phase B). Separation of the sample was executed with a 120-min gradient at a 300 nL/min flow rate. Gradient B was as follows: 8–35% for 92 min, 35–45% for 20 min, 45-100% for 2 min, 100% for 2 min, 100–2% for 2 min and 2% for 2 min. Data-dependent acquisition was performed in the profile and positive mode with the Orbitrap analyzer at a resolution of 70,000 (200 m/z) and m/z range of

350–1400 for MS1; for MS2, the resolution was set to 17,500 (200 m/z). The automatic gain control (AGC) target was set to 1.0e+06 for MS1 and 1.0e+05 for MS2. The top 10 most intense ions were fragmented by higher energy collisional dissociation (HCD) with a normalized collision energy (NCE) of 28% and isolation window of 2 m/z. The dynamic exclusion time window was 30 s.

MaxQuant database search

Raw MS files were processed with MaxQuant (Version 1.5.6.0). The human protein sequence database (Uniprot_HUMAN_2016_09) was downloaded from UNIPROT. This database and its reverse decoy were then compared by MaxQuant software. The quantification type was MS1; trypsin was set as the specific enzyme with up to 2 miss-cleavages; oxidation [M] and acetyl [protein N-term] were considered as variable modifications, whereas carbamidomethyl [C] was set as a fixed modification; and both peptide and protein FDR should be less than 0.01. Only unmodified unique peptides were used for quantification. iBAQ label-free quantification was also performed with log fit checked.

Figure legend

Supplemental information includes 4 figure and 1 table.

Supplementary Table

Supplementary Table 1 The clinical characteristics of unaffected and diabetic patients (with and without diabetic foot) cohorts.

Supplementary Figure

Supplementary Figure 1. Bmp9 promote proliferation of keratinocytes.

(**A**) A CCK8 analysis was performed to evaluate the proliferative rate after exogenous Bmp9 stimulation (50pg/ml) in HDFs.

(**B**) A CCK8 analysis was performed to evaluate the proliferative rate after exogenous Bmp9 stimulation (50pg/ml) in HaCaTs.

Supplementary Figure 2. BMP9 remodels cytoskeleton in human dermal fibroblast (HDF) cells.

(**A-B**) Gene set enrichment analysis (GSEA), gene expression profiles associated to cytoskeleton remodeling were enriched in BMP9-treated HDFs relative to control cells

(**C**) Label Free MS revealed proteins that assist cell motility through the regulation of cytoskeleton, including MYOSIN6, α-SMA, TUBB2B, STMN2 were identified to be up-regulated after BMP9 stimulation.

(**D**) The protein levels of MYOSIN6, α-SMA, TUBB2B and STMN2, Collagen 1 and fibronectin were measured by Western Blot in both HDF and HaCaT cells.

(**E**) An immunofluorescence of MYOSIN6, α-SMA and Collagen 1 were performed after stimulation of BMP9 in HDFs.

(**F**) A western blot was performed to measure the microtubule protein in soluble

supernatant and cytoskeleton after BMP9 stimulation

(**G**) An immunofluorescence of stress fiber of F-actin displayed sharp reduction in fibroblasts treated with BMP9.

Supplementary Figure 3. Application of Bmp9-ointment promotes wound healing in vivo.

(**A**) A skin injury model with a 6 mm-diameter-punch in *Bmp9* knockout mice was established after reimbursement of Bmp9-ointment and control-ointment. n=5 for each group.

(**B**) A H-E staining of wound area in Bmp9-/- mice after 2 days.

(**C**) Image of wound area in Bmp9-/- mice after reimbursement of Bmp9-ointment (**left**) and control protein ointment (**right**).

(**D**) A skin injury model with a 6 mm-diameter-punch in both WT mice was established after reimbursement of Bmp9-ointment and control-ointment. n=5 for each group.

(**E**) A H-E staining of wound area in WT mice after 2 days.

(**F**) Image of wound area in WT mice after reimbursement of Bmp9-ointment (**left**) and control protein ointment (**right**).

Supplementary Figure 4. Schematic of the study. Secreted cytokine BMP9 is essential for wounding healing.

BMP9 promotes wounding healing by regulating fibroblast and keratinocyte migration and proliferation through Smad signaling and cytoskeleton remodeling. BMP9 specifically bind to the auxiliary receptor endoglin and the serine threonine receptor ALK1, thus triggering Smad1,5 phosphorylation and promoted ID1/2

expression. Also, BMP9 reshaped cytoskeleton to a more flexible form, with increased extracellular matrix secretion.

Supplementary Table 1. The clinical characteristics of patient cohorts.

Features	Diabetic Foot (DF)	Type 2 Diabetes (T2D)	Type 1 Diabetes (T1D)	Unaffected
n	19	17	17	34
Sex, F/M	11/8	6/11	10/7	17/17
Age	59.4±16.4	47.3±19.65	68.4±18.2	28.4±8.2
HbA1c (%)	9.69±1.02	8.54±0.57	9.06±0.77	NA
FBG (mmol/L)	9.15 ± 4.33	8.07 ± 2.75	9.66± 3.61	NA
2hOGTT(mmol/L)	17.93 ± 4.13	13.85 ± 8.72	17.85 ± 8.11	NA

Supplementary Figure1

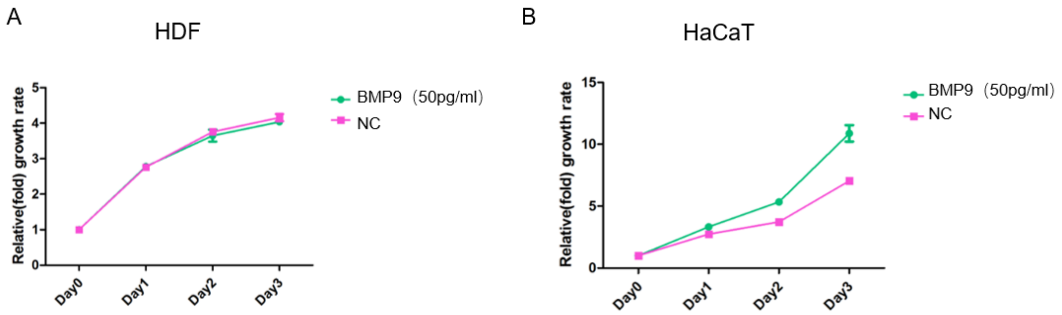

Supplementary Figure 2

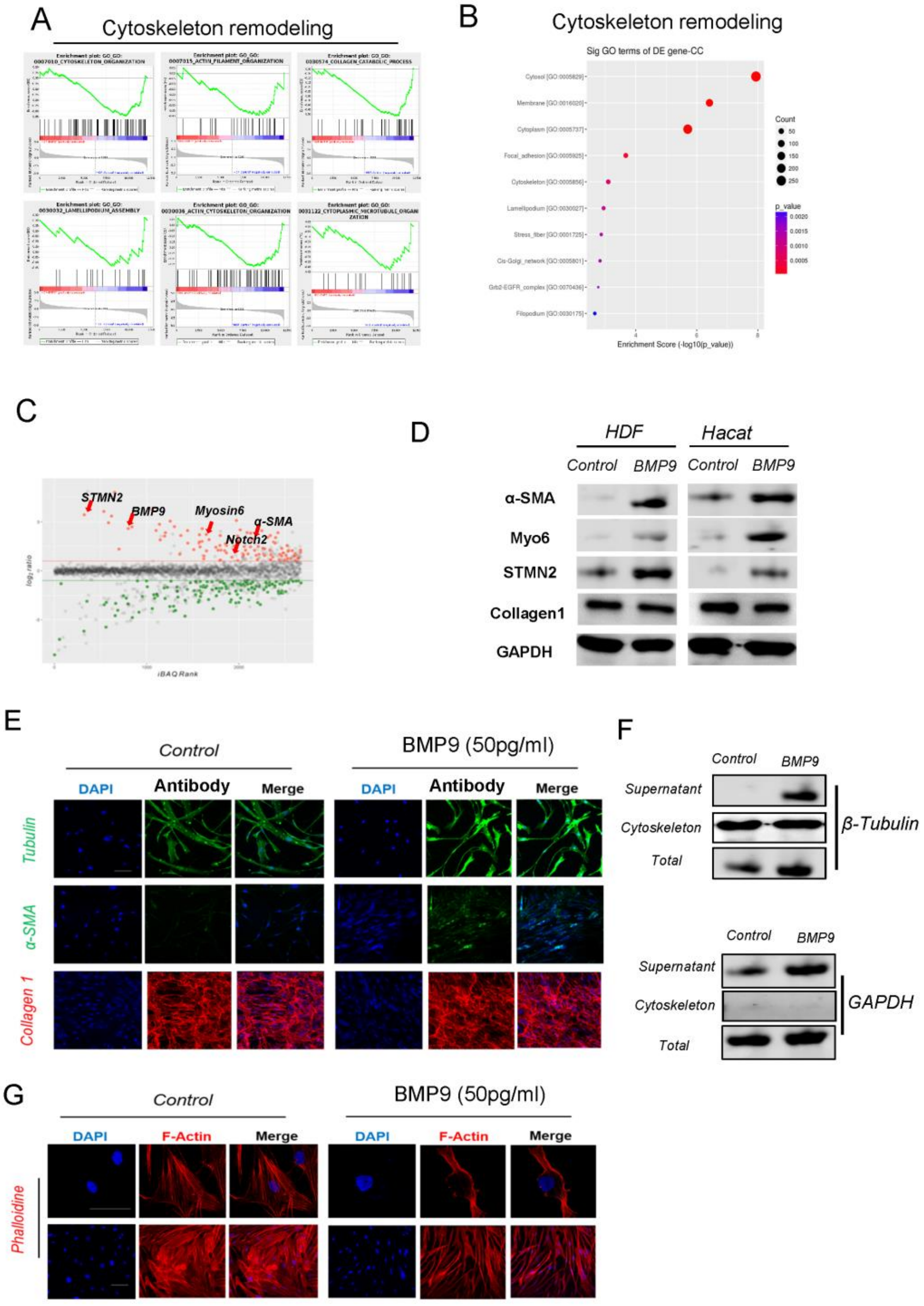

Supplementary Figure 3

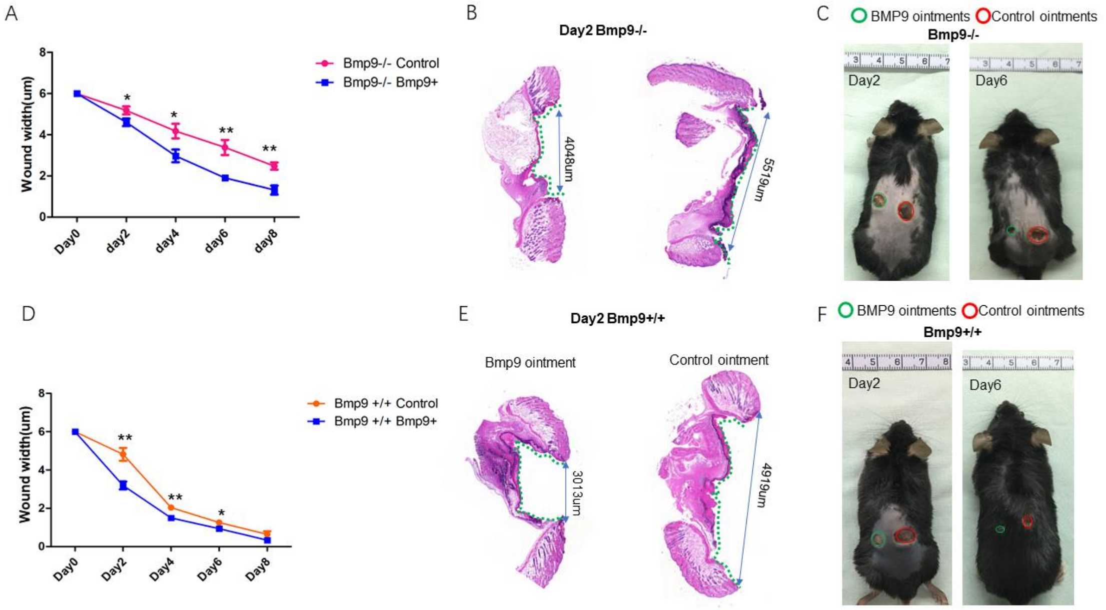

Supplementary Figure 4

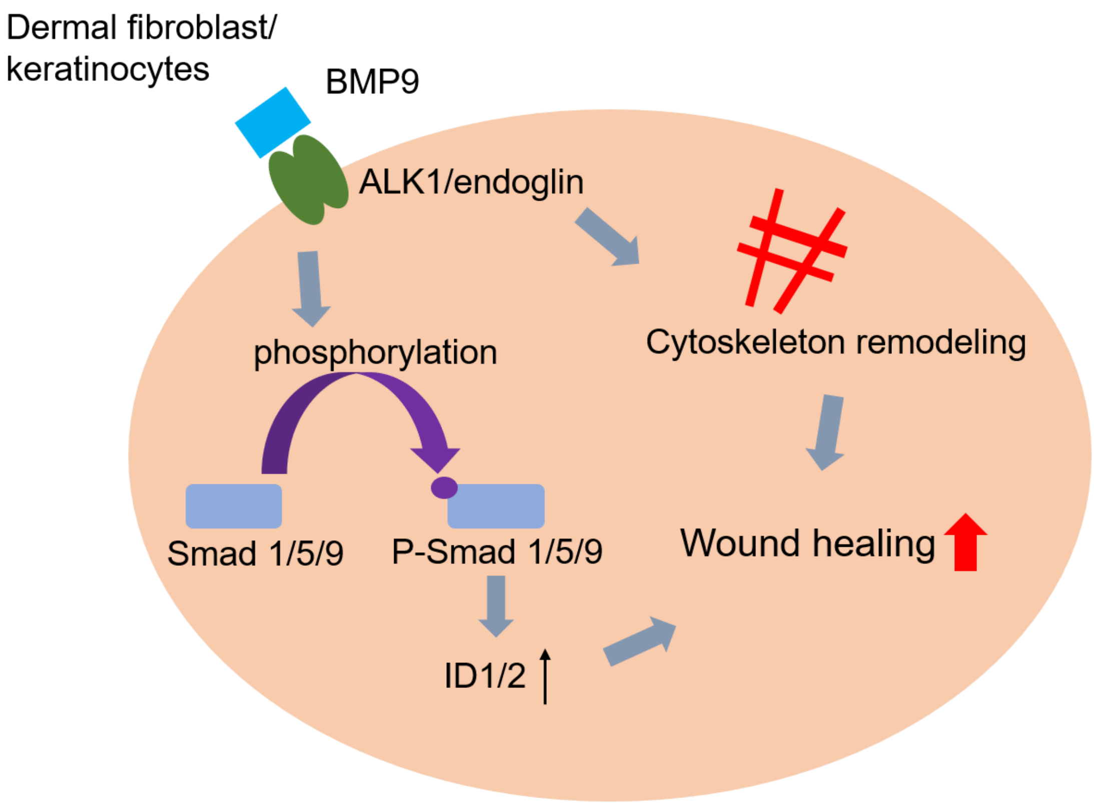

Reference

1. Yang Z, Li P, Shang Q, Wang Y, He J, Ge S, et al. CRISPR-mediated BMP9 ablation promotes liver steatosis via the down-regulation of PPARalpha expression. Sci Adv. 2020;6(48).

2. Li Y, Shang Q, Li P, Yang Z, Yang J, Shi J, et al. BMP9 attenuates occurrence of venous malformation by maintaining endothelial quiescence and strengthening vessel walls via SMAD1/5/ID1/alpha-SMA pathway. J Mol Cell Cardiol. 2020;147:92-107.
